# Development and validation of a continuous metabolic syndrome severity score in the Tehran Lipid and Glucose Study

**DOI:** 10.1038/s41598-023-33294-w

**Published:** 2023-05-09

**Authors:** Mohammadjavad Honarvar, Safdar Masoumi, Ladan Mehran, Davood Khalili, Atieh Amouzegar, Fereidoun Azizi

**Affiliations:** 1 Endocrine Research Center, Research Institute for Endocrine Sciences, Shahid Beheshti University of Medical Sciences, Tehran, Islamic Republic of Iran; 2 Department of Biostatistics, Faculty of Medical Sciences, Tarbiat Modares University, Tehran, Islamic Republic of Iran; 3 Prevention of Metabolic Disorders Research Center, Research Institute for Endocrine Sciences, Shahid Beheshti University of Medical Sciences, Tehran, Islamic Republic of Iran; 4 Department of Biostatistics and Epidemiology, Research Institute for Endocrine Sciences, Shahid Beheshti University of Medical Sciences, Tehran, Islamic Republic of Iran

**Keywords:** Metabolic syndrome, Epidemiology

## Abstract

Metabolic syndrome (MetS), defined as the coexistence of interrelated cardiometabolic risk factors, is limited by ignoring the severity of the disease and individuals with a pre-metabolic state. We aimed to develop the first age- and sex-specific continuous MetS severity score in the adult population using confirmatory factor analysis (CFA) based on the MetS components in the Middle East. Using data from the population-based Tehran Lipid and Glucose Study (TLGS) I and II datasets, we conducted CFA of the single factor MetS on 8933 adults (20–60 years old) totally, and in age and sex subgroups. We allowed for different factor loadings across the subgroups to formulate age- and sex-specific continuous MetS severity score equations. Thereafter, we validated these equations in the dataset of TLGS III participants. Triglyceride had the highest factor loading across age and sex subgroups, indicating the most correlation with MetS. Except for women aged 40–60 years, waist circumference was the second most significant factor contributing to MetS. Systolic blood pressure was more closely related to MetS in women than in men. Systolic blood pressure and fasting plasma glucose had the weakest correlation with MetS among the 40–60 age group. Moreover, as women age, the contribution of fasting plasma glucose to MetS tended to decline, while it remained relatively constant in men. The resulting MetS severity score was correlated with age and homeostasis model assessment of insulin resistance. Furthermore, the continuous MetS severity score well predicted the traditional MetS according to receiver operating characteristic analysis in the validation dataset. The age- and sex-specific continuous MetS severity score for the West Asian adult population provides a tangible quantitative measure of MetS enabling clinicians to screen and monitor the individuals at risk and assess their metabolic trends.

## Introduction

Metabolic syndrome (MetS) is defined by a cluster of interrelated risk factors for cardiovascular disease (CVD) and type 2 diabetes mellitus (T2DM). Diagnosis of MetS is made through the presence of three of the five risk factors, namely, central obesity, elevated fasting glucose, triglycerides, and blood pressure, and low high-density lipoprotein cholesterol (HDL-C). MetS is predictive of major health outcomes by holding a two- and five-fold increased risk for CVD and T2DM, respectively^1,2^. Insulin resistance, inflammation, and neurohormonal activities might play an imperative role in the MetS pathophysiology and its transition to adverse health events through complex yet not fully understood mechanisms^3^.

The dichotomous nature of the traditional MetS criteria (presence vs. absence) contributes to several limitations. The value of Mets components varies across different age, sex, and ethnicity groups regarding their prevalence and related risk for adverse events (e.g., low prevalence of MetS and high risk of CVD in non-Hispanic blacks, and high prevalence of MetS in the elderly associated with low attributed risk for mortality have been reported)^4–6^. Moreover, traditional MetS criteria cannot identify individuals with pre-metabolic syndrome (preMetS) which its association with the increased risk of T2DM and CVD has been reported^7–9^; a continuous MetS severity score (cMetS-S) is able to include quantitative measures of all MetS components for the general population. In addition, the lack of a universal definition for MetS severity leads to lower precision in the calculation of future risks for adverse health events. Last but not least, monitoring the health status of patients with MetS is difficult without such measurements for severity.

To overcome these limitations, some investigators have defined the MetS severity score as the sum of z-scores of the MetS components^10^. With the use of confirmatory factor analysis (CFA), Gurka et al.^11^ took a step further by considering the correlation of these components as an indicator of the underlying processes involved in MetS, as well as the variation of this correlation by sex (male/female) and race (Hispanic, Non-Hispanic Black, and Non-Hispanic White). MetS severity scores were previously developed using CFA in the American (White, Hispanic, Black races), Korean, Mexican, and Singaporean adults^11–14^. However, no MetS severity score has been created for the adult population in Western Asia.

The current study aimed to develop the first age- and sex-specific MetS severity score in West Asia. We used CFA on data from the population-based Tehran Lipid and Glucose Study (TLGS) to evaluate how the five traditional MetS components cluster together as MetS by considering their weighted contribution. In addition to predicting the risk of future cardiometabolic diseases more precisely, this continuous score can be used to monitor the population at risk and the effect of lifestyle and medical interventions.

## Method

### Study population and design

Data were obtained from the Tehran Lipid and Glucose Study (TLGS), a longitudinal 20-year cohort performed to observe non-communicable diseases in Iran, a country in West Asia. This ongoing cohort study consists of six checkups (TLGS I-VI) with 3-year follow-up intervals. To develop cMetS-S equations using CFA, we used the dataset of participants recruited in the TLGS I and II, and to validate these findings, we used the dataset of the TLGS III. To formulate the cMetS-S, we excluded individuals with known or unknown diabetes (fasting plasma glucose > 125 mg/dl), anti-diabetic, anti-hypertensive, or anti-hyperlipidemic medication, history of CVD, missing data, extreme levels of any MetS components (below − 3SD and above + 3SD) and pregnant women.

### Data collection

In each checkup, data of the participants were collected through interview-based questionnaires and examinations by trained physicians. Blood pressure was measured twice using a sphygmomanometer after 15 min rest in a sitting position, and the average systolic and diastolic blood pressure were recorded accordingly. Waist circumference, height, and weight were measured to the nearest 0.1 cm, 0.1 cm, and 0.1 kg, respectively. Venous blood samples were drawn after 12-h overnight fasting; serum insulin, plasma glucose, triglycerides, and total and HDL cholesterol were measured by the standard protocols and equipment^15^. MetS was defined as the presence of at least three of the MetS components: (1) central obesity defined as waist circumference ≥ 90 cm in both sexes according to the population-specific cutoff presented by the national committee^16^ (2) high fasting plasma glucose (≥ 100 mg/dl) or anti-diabetic medication (3) low HDL-C (< 40 mg/dl for men, and < 50 mg/dl for women) (4) hypertriglyceridemia (≥ 150 mg/dl) or the specific drug treatment (5) high blood pressure defined by systolic blood pressure ≥ 130 mmHg, diastolic blood pressure ≥ 85 mmHg, or treatment with medications of hypertension^17^.

### Statistical analysis

We performed CFA on the five identified MetS components: waist circumference, fasting plasma glucose, systolic blood pressure, triglycerides, and HDL-C to consider the weighted contribution of these components to the unobserved latent variable of MetS. Several CFA were performed on the eligible TLGS participants aged 20–60 years, and the results were presented in total and on age- (20–39 years and 40–60 years) and sex-specific basis.

To perform CFA analysis, since systolic and diastolic blood pressure are highly correlated^18^, we chose to only include systolic blood pressure in CFA as it is more strongly associated with insulin resistance^19^. The inverse of HDL value was used to interpret higher factor loading similarly to other MetS factors. The triglyceride's value was naturally log-transformed due to its skewed distribution. All the five MetS components in the models were standardized at mean = 0 and SD = 1 over the entire sample. One-factor model CFA was performed, and it was assumed that the measurement errors of the five components were not correlated. The factor loadings were indicative of the magnitude of the association between each component and the unobserved latent variable of MetS. The factor loadings > 0.3 were considered to show a moderate correlation. Models were developed in the total population and age and sex subgroups with and without the assumption of the equality of factor loadings across the age and sex subgroups, respectively. Factor scores were produced using proper linear combinations of the variables. We calculated factor scores and cMetS-S using linear regression analysis with unstandardized MetS components to allow for potentially higher standardized scores within sex and age groups. The standardized factor coefficients in the final models were applied to calculate the cMetS-S for each individual. For ease of use in clinical settings, the MetS components and their factor coefficients obtained from the CFA have been back-transformed so that actual values of MetS components can be placed in the equations. The resulting cMetS-S score can be standardized in each population and interpreted as z-scores (mean = 0; SD= 1), with greater values representing higher MetS severity.

The performance of the overall and age- and sex-specific models was compared with various fit indices. To evaluate the model fitness, the chi-squared test, the root mean square error of approximation (RMSEA; a good fit < 0.05), standardized root mean square residual (SRMR; good fit < 0.08), comparative fit index (CFI, good fit > 0.90), the goodness of fit index (GFI, good fit > 0.90), Bentler-Bonett normed fit index (NFI, good fit > 0.90), and Akaike's information criterion (AIC, smaller values indicates a better fit) were used. External validation was performed on the participants in TLGS III (the validation dataset) by receiver operating characteristic (ROC) analysis to evaluate the overall predictive performance of the resulted cMetS-S for the traditional MetS criteria. The area under the curve (AUC) value of 0.90 or higher was considered very accurate. We inspected the correlation between the cMetS-S and factors such as age and natural logarithm of homeostasis model assessment of insulin resistance (HOMA-IR) on the validation dataset, only excluding participants with extreme MetS outliers. The linear regression was fit using a natural log-transformed value of HOMA-IR in participants with available data regarding HOMA-IR. In addition, the mean value of the cMetS-S was determined in the groups based on the number of MetS components for exploratory purposes.

### Ethical approval

This study was performed according to the ethical principles of the Helsinki Declaration and approved by the National Research Council of the Islamic Republic of Iran (IR.SBMU.ENDOCRINE.REC.1401.066), the Human Research Review Committee of the Endocrine Research Center, Shahid Beheshti University, Tehran, Iran. Written informed consent was obtained from all subjects.

## Results

The sample of participants used for the series of CFA consisted of 8933 individuals aged 20–60 in the TLGS I and II datasets with complete data for MetS components. The mean values of age, waist circumference, systolic blood pressure, triglyceride, and fasting plasma glucose differed notably across age and sex groups. The model fit indices in the developed models, including RMSEA, SRMR, GFI, NFI, and CFI, were 0.09–0.15, 0.039–0.070, 0.85–0.98, 0.93–0.98, and 0.75–0.92, respectively (Table 1). The factor loadings of the MetS components varied by age and sex groups. Among the MetS components, triglyceride had the highest factor loading (0.73–84) in all subgroups. Waist circumference had the second highest factor loading value except in women aged 40–60. Fasting plasma glucose and systolic blood pressure had the lowest factor loading among the MetS components, the lowest being in the age range 40–60 years. Systolic blood pressure exhibited higher factor loadings in women compared to the corresponding subgroups of men. The factor loading of fasting plasma glucose was consistent between men aged 20–39 years and those aged 40–60 years. However, in women, the factor loading value of fasting plasma glucose was higher among those aged 20–39 years, as compared to women aged 40–60 years.

**Table 1:** Model fit indices and factor loadings in sex and age subgroups.

	Total	Men			Women		
	20–60 years	20–60 years	20–39 years	40–60 years	20–60 years	20–39 years	40–60 years
No of participants	8933	3794	2451	1343	5139	3393	1746
Age (years)	34.60 ± 10.36	35.39 ± 10.33	29.93 ± 6.05	48.44 ± 5.70	33.88 ± 10.34	28.37 ± 5.74	47.54 ± 5.27
WC (cm)	87.76 ± 12.40	90.60 ± 11.27	89.59 ± 11.46	92.96 ± 10.46	85.04 ± 12.83	81.76 ± 11.54	93.02 ± 12.32
SBP (mmHg)	111.03 ± 14.26	114.39 ± 13.40	112.77 ± 11.47	118.23 ± 16.55	108.02 ± 14.35	104.38 ± 11.10	116.99 ± 17.24
HDL-C (mg/dL)	40.14 ± 11.04	36.24 ± 9.04	36.30 ± 8.98	36.09 ± 9.19	43.64 ± 11.50	43.67 ± 11.64	43.57 ± 11.15
TG (mg/dL)	146.49 ± 104.3	171.46 ± 126.28	162.34 ± 122.33	193.21 ± 132.96	124.09 ± 72.69	111.68 ± 66.38	154.83 ± 78.44
FPG (mg/dL)	88.71 ± 8.92	89.97 ± 8.86	88.89 ± 8.16	92.53 ± 9.89	87.57 ± 8.83	85.60 ± 7.44	92.47 ± 10.04
Model fit indices							
Chi-square	664.337	318.657	116.244	150.051	345.620	117.501	105.298
AIC	93,110.060	36,928.139	22,449.751	13,961.174	55,250.180	33,760.931	19,683.126
RMSEA (95% CI)	0.13 (0.12,0.14)	0.14 (0.13,0.15)	0.11 (0.09,0.12)	0.15 (0.13,0.17)	0.13 (0.12,0.14)	0.09 (0.08,0.11)	0.12 (0.10,0.14)
SRMR	0.051	0.059	0.046	0.07	0.049	0.039	0.058
GFI	0.960	0.957	0.978	0.847	0.967	0.985	0.975
NFI	0.955	0.948	0.970	0.934	0.980	0.966	0.960
CFI	0.87	0.84	0.90	0.75	0.88	0.92	0.80
Factor loadings							
WC	0.63	0.58	0.57	0.48	0.64	0.59	0.30
SBP	0.44	0.31	0.30	0.22	0.50	0.39	0.25
HDL-C	0.42	0.45	0.48	0.47	0.35	0.45	0.46
TG	0.73	0.75	0.79	0.74	0.71	0.73	0.84
FPG	0.39	0.31	0.27	0.26	0.43	0.35	0.23

*AIC* Akaike information criterion, *RMSEA* root mean squared error of approximation, *CI* confidence interval, *SRMR* standard root mean square residual, *GFI* the goodness of fit index, *NFI* Bentler–Bonett normed fit index, *CFI* comparative fit index, *WC* waist circumference, *SBP* systolic blood pressure, *HDL-C* high-density lipoprotein cholesterol, *TG* triglyceride, *FPG* fasting plasma glucose.

The final equations resulted from the CFA in each group are presented in Table 2. Nine equations were developed totally, in sex and age subgroups of 20–60, 20–39, and 40–60 years. The resulting equations can show traditional MetS criteria on a continuous scale, while representing the MetS severity. Since MetS components contribute to MetS differently according to age groups and sex, the age- and sex-specific equations are recommended to be used in further studies (highlighted equations in Table 2). The cMetS-S of each individual can be obtained with data on sex, age, and the five MetS components using the age- and sex-specific formulas. The age- and sex-specific cMetS-S value ranged from − 0.86 to 1.16. The cMetS-S range of value for each sex and age subgroup is presented in Table 3. As an example, the cMetS-S of four random participants were calculated for the given MetS components values using these formulas in Supplementary Table S1.

**Table 2:** Age and sex-specific continuous metabolic syndrome severity score (cMetS-S) equations derived from the confirmatory factor analysis.

	Age (years)	Equations
Men	**20–39**	**− 1.79 + 0.0016 × SBP + 0.0045 × WC + 0.0017 × FPG + 0.24 × ln (TG) − 0.0042 × HDL-C**
	**40–60**	**− 1.67 + 0.0007 × SBP + 0.0034 × WC + 0.0014 × FPG + 0.25 × ln (TG) − 0.0042 × HDL-C**
	20–60	− 2.28 + 0.0019 × SBP + 0.0067 × WC + 0.0027 × FPG + 0.28 × ln (TG) − 0.0054 × HDL-C
Women	**20–39**	**− 2.43 + 0.0039 × SBP + 0.0066 × WC + 0.004 × FPG + 0.28 × ln (TG) − 0.0052 × HDL-C**
	**40–60**	**− 2.37 + 0.001 × SBP + 0.0021 × WC + 0.0015 × FPG + 0.41 × ln (TG) − 0.004 × HDL-C**
	20–60	− 4.13 + 0.0065 × SBP + 0.012 × WC + 0.007 × FPG + 0.39 × ln (TG) − 0.006 × HDL-C
Total	20–39	− 2.34 + 0.003 × SBP + 0.0061 × WC + 0.0032 × FPG + 0.29 × ln (TG) − 0.0055 × HDL-C
	40–60	− 1.94 + 0.0006 × SBP + 0.0019 × WC + 0.0011 × FPG + 0.33 × ln (TG) − 0.003 × HDL-C
	20–60	− 3.39 + 0.0044 × SBP + 0.0099 × WC + 0.0054 × FPG + 0.36 × ln (TG) − 0.0063 × HDL-C

The age- and sex-specific equations are marked in bold.

*SBP* systolic blood pressure, *WC* waist circumference, *FPG* fasting plasma glucose, *TG* triglyceride, *HDL-C* high-density lipoprotein cholesterol.

**Table 3:** The range of age- and sex-specific continuous metabolic syndrome severity score (cMetS-S) value in the TLGS population by sex and age category.

Sex	Age (years)	cMetS-S	
		Min	Max
Male	20–39	− 0.65	0.75
	40–60	− 0.65	0.70
Female	20–39	− 0.7	1.16
	40–60	− 0.86	1.10

*cMetS-S* continuous metabolic syndrome severity score, *TLGS* Tehran Lipid and Glucose Study.

**Figure 1:**
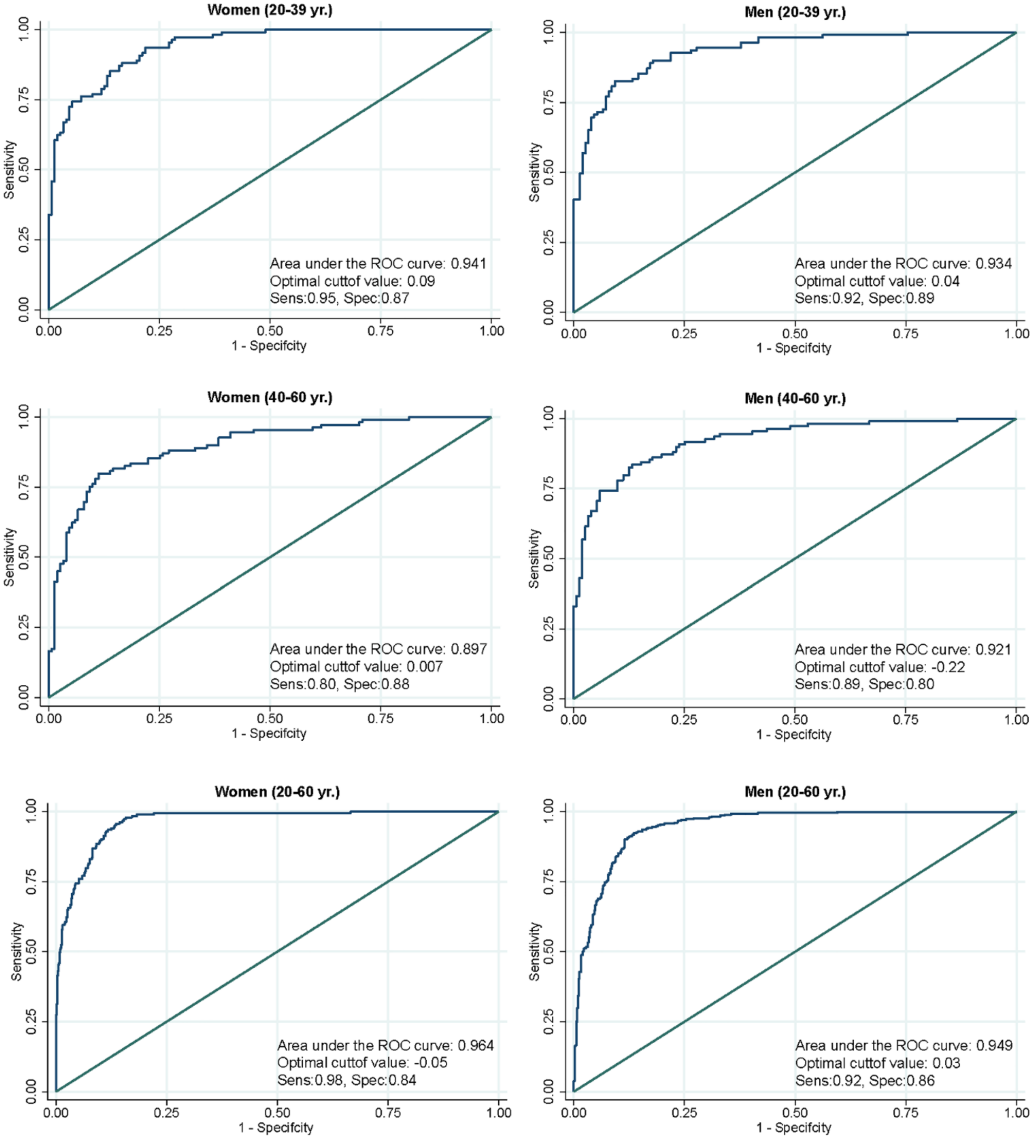
ROC analysis of sex-and age-specific continuous metabolic syndrome severity score (cMetS-S) for prediction of MetS. The ROC analyses of cMetS-S equations are presented, demonstrating the ROC curve of the cMetS-S equations in women (left pane) and men (right pane) in age categories of 20–39, 40–60, and 20–60 years old. *ROC* receiver operating characteristic.

**Figure 2:**
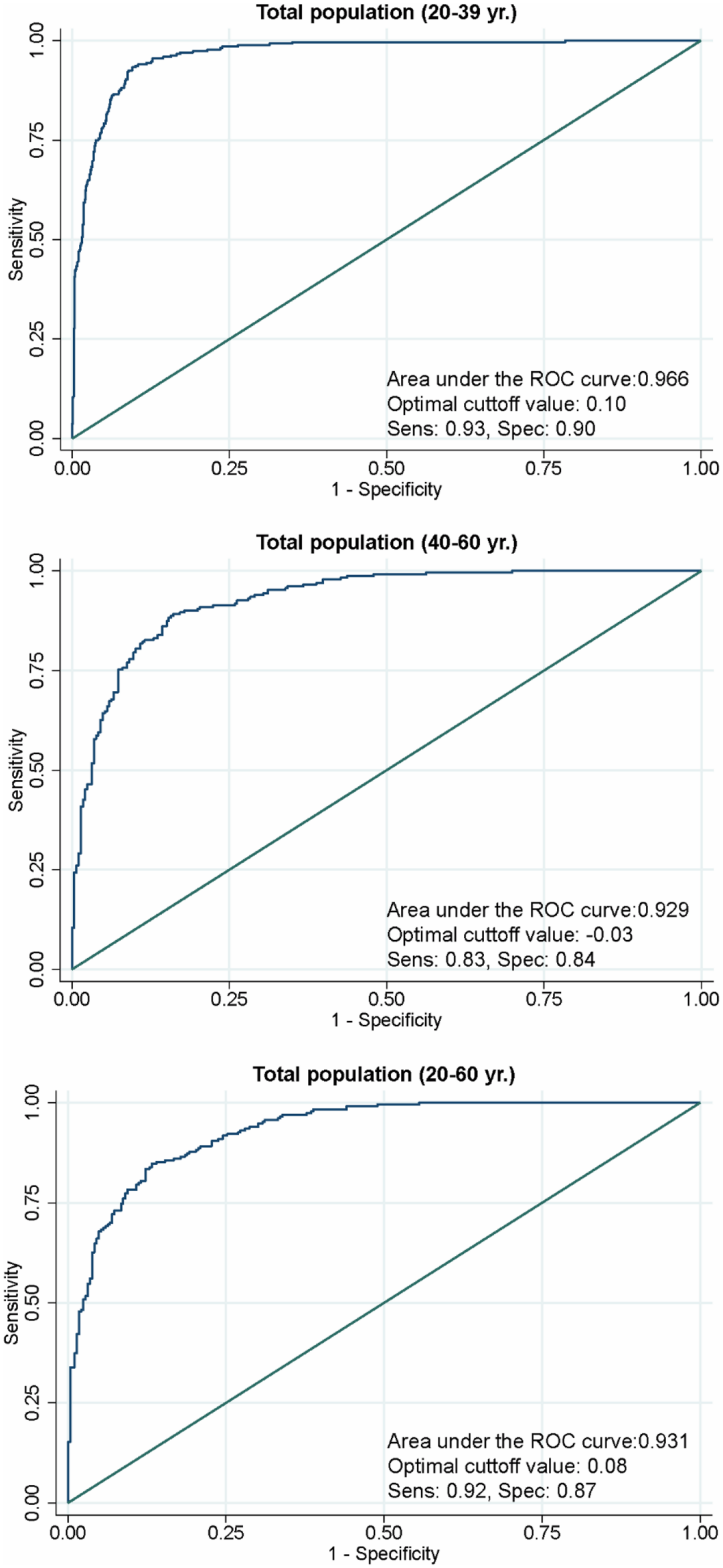
ROC analysis of age-specific continuous metabolic syndrome severity score (cMetS-S) for prediction of MetS. *ROC* receiver operating characteristic.

**Figure 3:**
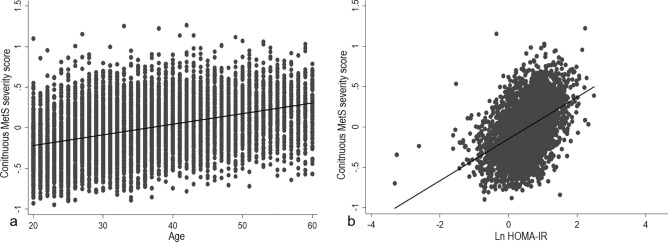
Scatter plots representing the correlation of continuous metabolic syndrome severity score (cMetS-S) with age (**a**) and Ln HOMA-IR (**b**). *HOMA-IR* homeostasis model of insulin resistance.

To externally validate the cMetS-S, we utilized the data of participants in TLGS III. The ROC analysis of the models demonstrated an excellent diagnostic performance for traditional MetS classification. Except for the women aged 40–60 years, all derived cMetS-S had area under the curve (AUC) values > 0.90 (Figs. 1, 2). The highest AUC value for the age- and sex-specific cMetS-S was in women aged 20–39 years (AUC = 0.941), and the lowest AUC value belonged to women aged 40–60 (AUC = 0.897) (Fig. 1). The concordance index (C-statistic) values in all groups were higher than 0.90 (Table 4). The cMetS-S was also linearly correlated with age and the natural logarithm of HOMA-IR values (*p* < 0.001) (Fig. 3).

**Table 4:** Concordance index of the age- and sex-specific models in the external validation dataset.

	Age group (y)	C- statistics	Hosmer and Lemeshow test
Men	20–39	0.93	< 0.001
	40–60	0.94	< 0.001
	20–60	0.93	< 0.001
Women	20–39	0.96	< 0.001
	40–60	0.94	0.026
	20–60	0.93	< 0.001
Total	20–39	0.96	0.048
	40–60	0.91	< 0.001
	20–60	0.93	< 0.001

**Figure 4:**
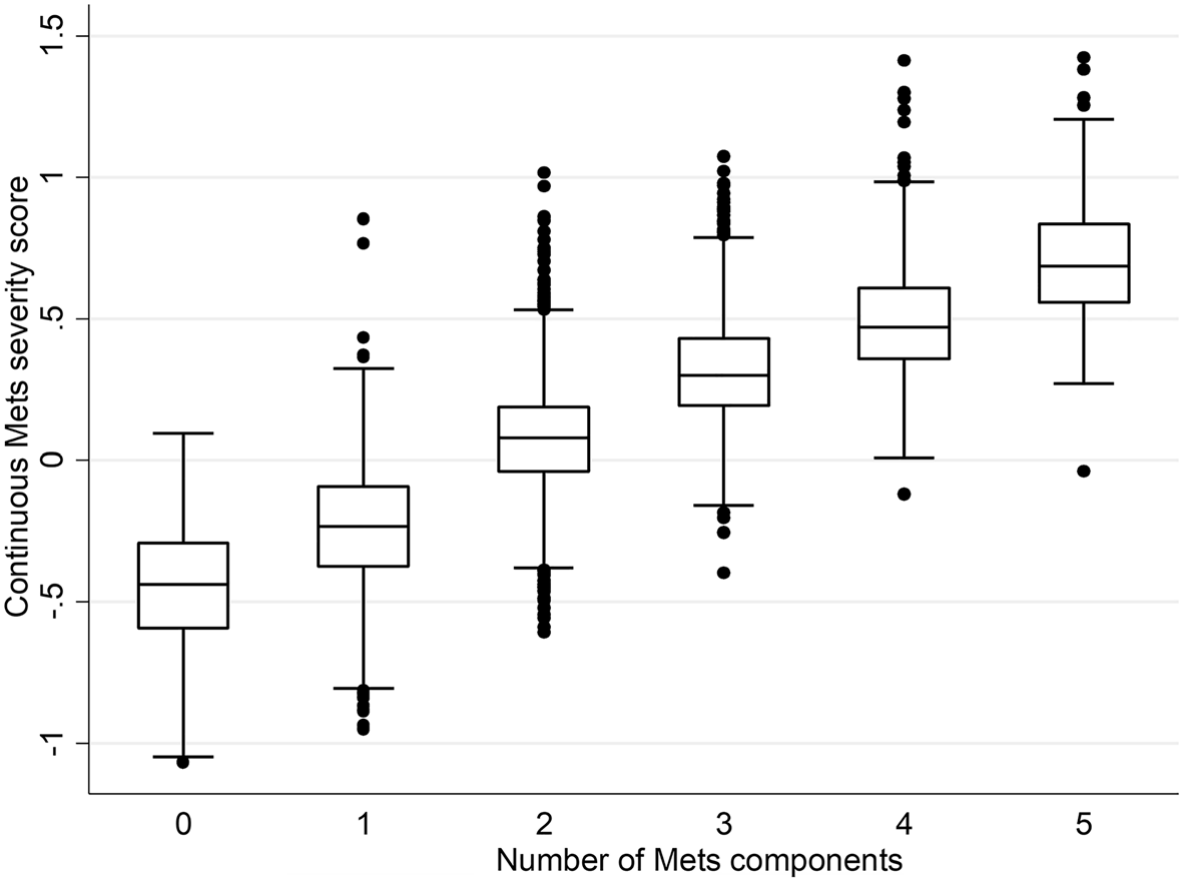
Mean, median and interquartile range of continuous metabolic syndrome severity score (cMetS-S) by the number of MetS traits.

Figure 4 shows the mean values of the cMetS-S derived from the total population in the subgroups based on the number of MetS components. Individuals with less than two MetS components had a mean cMetS-S value below zero. This value increased by adding to the number of MetS components; individuals with two, three, four, and five MetS components had average cMetS-S of 0.07 ± 0.19, 0.31 ± 0.19, 0.49 ± 0.19, and 0.70 ± 0.23, respectively (Table 5).

**Table 5:** Mean values of continuous metabolic syndrome severity score (cMetS-S) based on the number of MetS components.

Number of MetS components	cMetS-S	
	Mean	SD
0	− 0.45	0.21
1	− 0.24	0.21
2	0.07	0.19
3	0.31	0.19
4	0.49	0.19
5	0.70	0.23
Total	0	0.37

*cMetS-S* continuous metabolic syndrome severity score.

Table 6 presents the studies which developed MetS severity equations based on CFA for adults. For each study, the factor loadings of the MetS components are displayed in descending order to demonstrate their respective contributions to MetS.

**Table 6:** Factor loadings derived from confirmatory factor analysis in studies developing MetS severity score equations.

Studies	The current study	Gurkha et al.^11^			Huh et al.^12^	Low et al.^13^	Pérez-Castro et al.*^14^
Country	Iran	United States of America			Korea	Singapore	Mexico
Ethnicity/region	West Asia	White	Black	Hispanic	Asia–Pacific	Asia–Pacific	Latin America
Sex-specific	✓	✓			✓	✓	✓
Age-specific	✓	✗			✓	✗	✗
MetS components sorted by factor loading values from highest to the lowest (values)							
Men	20–39 years	20–64 years	20–64 years	20–64 years	20–39 years	≥40 years	20–59 years
	①TG (0.79)	①TG (0.73)	①WC (0.67)	①TG (0.70)	①TG (0.68)	①HDL (0.76)	MetSx-WC
	②WC (0.57)	②HDL (0.60)	②HDL (0.64)	②HDL (0.57)	②WC (0.66)	②TG (0.72)	①TG (0.90)
	③HDL (0.48)	③WC (0.49)	③TG (0.45)	③WC (0.36)	③HDL (0.47)	③WC (0.55)	②HDL (0.78)
	④SBP (0.30)	④FPG (0.26)	④FPG (0.37)	④FPG (0.27)	④FPG (0.40)	④FPG (0.25)	③FPG (0.50)
	⑤FPG (0.27)	⑤SBP (0.17)	⑤SBP (0.16)	⑤SBP (0.19)	⑤SBP (0.37)	⑤SBP (0.17)	④SBP (0.23)
	40–60 years				40–60 years		
	①TG (0.74)				①TG (0.67)		
	②WC (0.48)				②WC (0.57)		
	③HDL (0.47)				③HDL (0.47)		
	④FPG (0.26)				④FPG (0.30)		
	⑤SBP (0.22)				⑤SBP (0.24)		
Women	20–39 years	20–64 years	20–64 years	20–64 years	20–39 years	≥40 years	20–59 years
	①TG (0.73)	①WC (0.71)	①WC (0.77)	①TG (0.59)	①WC (0.67)	①TG (0.72)	MetSx-WC
	②WC (0.59)	②TG (0.56)	②FPG (0.46)	②WC (0.45)	②TG (0.57)	②HDL (0.69)	①TG (0.95)
	③HDL (0.45)	③HDL (0.52)	③HDL (0.40)	②HDL (0.45)	③HDL (0.44)	③WC (0.54)	②FPG (0.69)
	④SBP (0.39)	④FPG (0.46)	④TG (0.37)	④FPG (0.44)	④FPG (0.43)	④FPG (0.34)	③HDL (0.66)
	⑤FPG (0.35)	⑤SBP (0.33)	⑤SBP (0.31)	⑤SBP (0.38)	⑤SBP (0.33)	⑤SBP (0.30)	④SBP (0.23)
	40–60 years				40–60 years		
	①TG (0.84)				①TG (0.69)		
	②HDL (0.46)				②WC (0.51)		
	③WC (0.30)				②HDL (0.51)		
	④SBP (0.25)				④FPG (0.34)		
	⑤FPG (0.23)				⑤SBP (0.28)		

*MetS* metabolic syndrome, *TG* triglyceride, *WC* waist circumference, *HDL-C* high-density lipoprotein cholesterol, *SBP* systolic blood pressure, *FPG* fasting plasma glucose.

*The study conducted by Pérez-Castro et al. considered MetS-WC (Metabolic syndrome-waist circumference) as the latent variable instead of MetS in the confirmatory factor analysis.

## Discussion

In the current study, we formulated the first age- and sex-specific cMetS-S for the West Asian adult population using CFA, considering the contributed weight of individual MetS components to MetS. Moreover, we externally validated the predictive ability of cMetS-S and also observed the correlation of this score with insulin resistance (HOMA-IR) as the hallmark of MetS. The value of MetS components differed by sex and age. Among the Mets components, triglyceride had consistently the highest correlation with MetS in all the subgroups. Waist circumference was the second highest contributing MetS component to MetS except in women aged 40–60 years. The correlation of systolic blood pressure and fasting plasma glucose with MetS was the lowest in the age group of 40–60 years. Systolic blood pressure had more correlation with MetS in women compared to men of the same age subgroup. Additionally, the contribution of fasting plasma glucose to MetS tended to decrease with age in women, while it remained nearly the same in men as they get older. Therefore, both the value and correlation of MetS components to MetS varied by sex and age, emphasizing the significance of employing age- and sex-specific cMetS-S.

The traditional MetS defined with at least three abnormal MetS components is limited by missing individuals with extreme values, or high borderline normal values of each MetS component. This definition leads to loss of data regarding the severity of abnormality of each component and missing individuals who might be at risk of future adverse health events that are not foreseen in the definition. The term preMetS has been proposed by some researchers to address such individuals because of its reported association with T2DM and CVD^8,9^. Several phenotypes of the preMetS have been defined with their new corresponding indices. In a recent meta-analysis, the hypertriglyceridemic-waist (HTGW) phenotype was reported to increase the risk of CVD independent of the established risk factors^20^. Triglyceride-glucose (TyG) index which represents a quantitative value of the high triglyceride and fasting plasma glucose phenotype of traditional metabolic clusters was associated with the risk of diabetes and cardio-cerebrovascular disease even after adjusting for other MetS components^21,22^. Moreover, the joint exposure of high triglyceride and fasting plasma glucose had an additive value for CVD prediction^23^. MetS severity score can not only identify individuals with MetS, but also provide cardiometabolic information on the individuals without MetS, and the ability to compare the related health risks within both populations.

In the current study, MetS severity score equations were developed in the total population, and on an age and sex-specific basis using CFA. MetS severity score of each individual with given data on age, sex, and the measures of the five MetS components can be calculated using these equations. The resulting score represents a continuous score, with its increasing value indicating higher MetS severity. Age- and sex-specific equations were only developed in the current and the Korean study by Huh et al.^12^ highlighting the effect of age on the correlation of MetS components with MetS. In the current study, triglyceride exhibited the highest factor loading (0.73–0.84), indicating the highest correlation with MetS; in other words, triglyceride might explain 53.29–70.56% (0.73^2^–0.84^2^) of the variance of the latent variable of MetS in the West Asian population in all age and sex subgroups. In the Korean study, triglyceride was also the highest correlated component to MetS except for women 20–39 years old. In line with the results of the Korean study, in the current study, the factor loading of each MetS component differed by sex and age, with the lowest factor loading observed for fasting plasma glucose and systolic blood pressure in all subgroups. The largest factor loadings for fasting plasma glucose and systolic blood pressure were present in women aged 20–39 years, suggesting the higher association of these components with MetS in this subgroup. In addition, the correlation of waist circumference with MetS was diminished in 40–60 year old adults in both studies regardless of sex. Our findings confirm the previous epidemiologic studies suggesting sex and age variation in the contribution of MetS components to MetS^24^. The difference in fat accumulation patterns, lipid metabolism, insulin resistance, and sex hormones are some potential explanations^25–28^. With advancing age, the prevalence of the MetS component and their contribution to MetS alter which may be due to the decrease in androgen levels in men, and the occurrence of menopause in women^5,29^.

Table 6 reviews the factor loadings of the MetS components in studies that formulated MetS severity scores using CFA in the adult population from different ethnicities/regions. Among the MetS components, triglyceride had the highest correlation with MetS as a latent variable in Iranian, Korean, and Hispanic populations, White American men, and Singaporean women. Waist circumference in White women, young Korean women and the Black population, and HDL-C in Singaporean men had the most significant correlation with MetS. Systolic blood pressure had the least contribution to MetS in most studies, showing the fact that the high rate of essential hypertension is independent of MetS^30^. The correlation of fasting plasma glucose with MetS varied by different ethnicities, with Black and Hispanic women having the highest, and Iranian middle-aged women having the least factor loadings. Although not all studies were exactly similar in terms of age and the corresponding subgroups, the weighted contribution of components to MetS showed variation by sex and ethnicity/region (Table 6). This finding aligns with the previous epidemiologic reports showing sex, ethnic and regional disparities in the prevalence of MetS components and their contribution to MetS^31–34^. Developed models from studies in Iran, Korea, and the USA were approximately similar in terms of fit indices values^11,12^. Although the studies conducted in Mexico^14^ and Singapore^13^ showed higher fit indices, they were limited by not excluding participants with CVD as the MetS endpoint, and those on antihypertensive, antihyperlipidemic, and anti-diabetic medications as the important confounders. Moreover, the MetS severity score in the Mexican population was developed using the international diabetes federation (IDF) MetS definition, with assumption of MetS-WC (MetS-waist circumference) and not MetS as the latent variable, making it different in the design.

Over the past 2 decades, various methods (e.g., counting and clustering the traits or sum of z-scores of the MetS components) have been proposed to define MetS severity to address some limitations regarding traditional MetS criteria; however, they all fail to reflect the severity of each MetS component and its weighted contribution to MetS. In the current study, MetS severity score was not defined on the outcome prediction basis (T2DM and CVD) and instead it was developed based on how MetS components cluster together as MetS by assigning weights to each MetS component using CFA. Previous studies have suggested MetS severity score derived from CFA is associated with T2DM and CVD independent of the individual MetS components^35–37^ which supports the clinical utility of MetS severity score.

This study is strengthened by the large population-based sample size and a well-designed analysis with the derivation of age- and sex-specific equations for the MetS severity score. External validation of the MetS severity score equations is another strength of this study. However, we could only evaluate the association of cMetS-S with insulin resistance (HOMA-IR) as the hallmark of Mets and data of other markers such as CRP, uric acid, and HbA1C was not available.

This study is the first to develop the age- and sex-specific MetS severity score with the excellent diagnostic ability for MetS using CFA in a West Asian adult population. Development of MetS severity score with such considerations provides a more tangible quantitative measure of MetS which enables clinicians to more precisely predict the incidence of T2DM and CVD, screen and monitor the individuals at risk, assess the metabolic trend and efficiency of any medical interventions if needed. As MetS severity score enables the quantitative measures of MetS in the general population, the development of this severity score for each ethnicity/region seems necessary.

## Supplementary Information

Supplementary Information.

## Data Availability

Datasets generated during and/or analyzed during the current study are not publicly available but are available from the corresponding author on reasonable request.

## Abbreviations

MetS: Metabolic syndrome
CVD: Cardiovascular disease
T2DM: Type 2 diabetes mellitus
HDL-C: High-density lipoprotein cholesterol
preMetS: Pre-metabolic syndrome
CFA: Confirmatory factor analysis
TLGS: Tehran Lipid and Glucose Study
cMetS-S: Continuous metabolic syndrome severity score
RMSEA: Root mean square error of approximation
SRMR: Standard root mean square residual
GFI: The goodness of fit index
NFI: Bentler–Bonett normed fit index
CFI: Comparative fit index
AIC: Akaike information criterion
ROC: Receiver operating characteristic
AUC: Area under the curve
HOMA-IR: Homeostasis model assessment of insulin resistance
HTGW: Hypertriglyceridemic-waist
IDF: International diabetes federation
