# Supplementary material for: Development and validation of a continuous metabolic syndrome severity score in the Tehran Lipid and Glucose Study
Source: Sci Rep. 2023 May 9;13:7529. doi: 10.1038/s41598-023-33294-w (PMC10170075; doi:10.1038/s41598-023-33294-w)
Supplement: Supplementary file 1 — Supplementary Information. [file 41598_2023_33294_MOESM1_ESM.pdf]

Development and Validation of a Continuous Metabolic Syndrome Severity Score in the Tehran Lipid and Glucose Study

Mohammadjavad Honarvar, MD[1]; Safdar Masoumi, PhD[1,2]; Ladan Mehran, MD, PhD [1] Davood Khalili, MD, PhD[3,4]; Atieh Amouzegar, MD[1]; Fereidoun Azizi, MD[1]

[1] Endocrine Research Center, Research Institute for Endocrine Sciences, Shahid Beheshti University of Medical Sciences, Tehran, I.R.Iran

[2] Department of Biostatistics, Faculty of Medical Sciences, Tarbiat Modares University, Tehran, I.R.Iran

[3] Prevention of Metabolic Disorders Research Center, Research Institute for Endocrine Sciences, Shahid Beheshti University of Medical Sciences, Tehran, I.R.Iran

[4] Department of Biostatistics and Epidemiology, Research Institute for Endocrine Sciences, Shahid Beheshti University of Medical Sciences, Tehran, I.R.Iran

Supplementary Table S1. Continuous metabolic syndrome severity score calculated for random participants using age- and sex-specific equations

Sex	Age (y)	SBP (mmHg)	WC (cm)	FPG (mg/dl)	TG (mg/dl)	HDL (mg/dl)	cMetS-S
Male	23	126	71	80	120	32	-0.12
Male	51	120	83	105	347	25	0.20
Female	30	120	102	104	354	39	0.57
Female	45	118	107	199	428	35	0.61

SBP, systolic blood pressure; WC, waist circumference; FPG, fasting plasma glucose; TG, triglyceride; HDL-C, high-density lipoprotein cholesterol; cMetS-S, continuous metabolic syndrome severity score
